# Combined Non-Target Effects of Insecticide and High Temperature on the Parasitoid *Bracon nigricans*

**DOI:** 10.1371/journal.pone.0138411

**Published:** 2015-09-18

**Authors:** Khaled Abbes, Antonio Biondi, Alican Kurtulus, Michele Ricupero, Agatino Russo, Gaetano Siscaro, Brahim Chermiti, Lucia Zappalà

**Affiliations:** 1 High Agronomic Institute of Chott-Mariem, University of Sousse, Tunisia, P.B. 47, 4042 Chott-Mariem, Sousse, Tunisia; 2 Dipartimento di Agricoltura, University of Catania, Alimentazione e Ambiente, via Santa Sofia 100, Catania, Italy; 3 Department of Environmental Science, Policy and Management, University of California, Berkeley, California, United States of America; 4 Department of Plant Protection, Agricultural Faculty, Cukurova University, Adana, Turkey; French National Institute for Agricultural Research (INRA), FRANCE

## Abstract

We studied the acute toxicity and the sublethal effects, on reproduction and host-killing activity, of four widely used insecticides on the generalist parasitoid *Bracon nigricans* (Hymenoptera: Braconidae), a natural enemy of the invasive tomato pest, *Tuta absoluta* (Lepidoptera: Gelechiidae). Laboratory bioassays were conducted applying maximum insecticide label rates at three constant temperatures, 25, 35 and 40°C, considered as regular, high and very high, respectively. Data on female survival and offspring production were used to calculate population growth indexes as a measure of population recovery after pesticide exposure. Spinetoram caused 80% mortality at 25°C and 100% at higher temperatures, while spinosad caused 100% mortality under all temperature regimes. Cyantraniliprole was slightly toxic to *B*. *nigricans* adults in terms of acute toxicity at the three temperatures, while it did not cause any sublethal effects in egg-laying and host-killing activities. The interaction between the two tested factors (insecticide and temperature) significantly influenced the number of eggs laid by the parasitoid, which was the lowest in the case of females exposed to chlorantraniliprole at 35°C. Furthermore, significantly lower *B*. *nigricans* demographic growth indexes were estimated for all the insecticides under all temperature conditions, with the exception of chlorantraniliprole at 25°C. Our findings highlight an interaction between high temperatures and insecticide exposure, which suggests a need for including natural stressors, such as temperature, in pesticide risk assessments procedures.

## Introduction

The use of natural enemies in agricultural eco-systems can be constrained by many factors related either to the biological features of these beneficials and/or to common agricultural practices, such as the use of broad spectrum insecticides, which is often identified as one of the key problems associated with the use of entomophagous insect species [1–3]. Indeed, chemical control is still one of the cornerstones of integrated pest management (IPM) tactics especially in highly valuable horticultural crops. In the case of tomato, a plethora of pesticides is often used to suppress pests and diseases associated with this crop in nurseries, greenhouse and open field [4]. Therefore, the evaluation of pesticide side effects on natural enemies is crucial for their effective inclusion in IPM programs [5–7].

Ecotoxicological risk assessment procedures are typically based on laboratory tests where organisms are exposed to toxicants under “standard conditions”. This implies that animals are kept under constant and optimal environmental parameters. However, organisms rarely experience optimal conditions in the field, where large fluctuations in climatic factors are the norm [8]. Moreover, because of large variability in climatic conditions among different regions of the world, results of tests carried out in standard conditions, can be valid for only a narrow strip of Earth where average climatic conditions resemble those used in the ecotoxicological tests. Assessing risks of single stressors might severely underestimate real side effects of pesticides [9]. Although synergistic interactions between the effects of various natural stressors (i.e. heat, cold, desiccation, oxygen depletion, pathogens and immuno-modulatory factors) and toxicants are not uncommon, the combined effect of natural stressors and chemical toxicants have been only seldom investigated [10]. These interactions may be complex, with resulting toxic effects that could be modified either through direct influence on organisms or by affecting chemical/biochemical pathways of the toxicants themselves [11].

Temperature is one of the most important natural factors, which is highly variable in the field and is of major importance for the physiology of an organism. For insects, temperature is the most important climatic factor governing the duration of their biological cycle, activity, reproduction and senescence [12–18]. For example, extreme temperatures can compromise the insect endosymbiont populations and, in several cases, differences between the thermal preferences of the host and those of its biocontrol agent cause a disruption of the temporal or geographical synchronization [19]. The impact of global warming is likely even more important in higher trophic levels that depend on the effect of these changes on the lower trophic levels. Therefore, primary and secondary parasitoids are organisms for which severe impacts of the temperature rise are expected [19]. Global warming is estimated to raise the average earth temperature by 1.5 to 5.8°C by the end of this century. This would have a wide range of drawbacks on both insect physiology, pesticides toxicity and insects’ response [20–21].

So far, only few ecotoxicological studies have been conducted on non-target insect species taking into account temperature variation [21–23]. However, the effect of temperature on insect susceptibility to pesticides was studied for various insect pests, and thus the relative efficacy of pesticides at various temperatures (e.g. [24–30]). The influence of temperature on toxicity can be either positive or negative depending on the mode of action of the insecticide, the insect species and the route of exposure (ingestion or contact). Temperature can alter the efficacy of pesticides by modifying their absorption, adsorption, persistence and disintegration. Most active ingredients showed synergistic effects with temperature and only in a few cases the highest toxicity was observed at intermediate temperature conditions [26,29,31]. Moreover, assessment of side effects of pesticides and other toxicants on non-target organisms has been traditionally based on acute mortality evaluation, mainly applying field recommended doses [6]. However, the proper assessment of sublethal effects, i.e. physiological and behavioral effects on individuals that survive exposure to a toxicant, is increasingly important when choosing new pesticides for IPM purposes [1,31–36].

Tomato is one of the most arable crops cultivated worldwide (more than 4.5 million hectares) with a global annual tomato production of about 164 million tones, 90% of which obtained in the Northern Hemisphere [37]. However, several key arthropod pests, can reduce the crop yield and/or increase the production costs. Among invasive tomato pests, *Tuta absoluta* (Meyrick) (Lepidoptera: Gelechiidae) caused extensive crop damage and disrupted the pre-existing IPM programs in the Western Palaearctic tomato crops [4,38]. Soon after, indigenous biocontrol agents, such as mirid predators and generalist parasitoids [39–43, and see Zappalà et al. [44] for a thorough review] were evaluated, with variable success, as sustainable tool to be included into IPM programs that also include pesticide applications [45–47].

Bearing this context in mind, we studied in the laboratory the acute toxicity and the sublethal effects, on reproduction, demographic traits and host-killing activity, of four insecticides (i.e., chlorantraniliprole, cyantraniliprole, spinosad and spinetoram) currently used in tomato crops. As non-target organism we used the generalist parasitoid *Bracon nigricans* Szépligeti (Hymenoptera: Braconidae), that was among the first natural enemies to adapt to *T*. *absoluta* in the Mediterranean basin [48–49]. Thus, laboratory bioassays were conducted at three constant temperatures, 25, 35 and 40°C, considered to be respectively regular, high and very high thermal regimes for tomato cropping systems.

## Material and Methods

### Insects

The owners of the fields where *T*. *absoluta* infested material was collected gave permission to conduct the study on their farms. The field studies did not involve endangered or protected species.

A colony of *B*. *nigricans* was established using individuals emerged from mature larvae of *T*. *absoluta* exposed to parasitism on sentinel tomato plants during a faunistic survey conducted in Sicily (Italy) in 2010 [48]. The colony was maintained in the laboratory using South American tomato leafminer larvae as hosts. The laboratory rearing of *T*. *absoluta* was initiated with wild individuals collected in 2009 from commercial tomato crops in Sicily (Italy). The moths were reared on potted tomato plants (Shiren variety) in laboratory conditions, and new wild individuals were introduced twice a year into the laboratory colony. Tomato seedlings were grown in small pots (0.3 liters), watered and fertilized following routine practices, and pesticide applications were strictly avoided. Tomato plants infested by mature South American tomato leafminer larvae (third and fourth instars) were obtained by releasing 40 South American tomato leafminer adults (1:1 sex ratio) on 10-40-cm high tomato plants inside 50 x 60 x 60 cm cages covered with a fine polyester mesh. The plants were transferred into parasitoid rearing plastic cages, on average, 14 d after the release of South American tomato leafminer adults, i.e. when the majority of the larvae had reached the third instar. New South American tomato leafminer-infested plants and honey droplets were supplied every 3 d into the parasitoid rearing cages. Newly emerged (<24h old) *B*. *nigricans* adults were obtained in 150-mm petri dishes ventilated by a 4-cm^2^ opening covered with a fine mesh net. Two females and four males were released into this arena, containing excised tomato leaves infested by mature South American tomato leafminer larvae and provided with honey droplets. After 48 h of exposure to parasitism, *B*. *nigricans* adults were removed and the infested parasitized material was maintained inside a climatic cabinet at 26±1°C, 50±10% R.H. and 14:10 L:D. Ten days later, newly emerged adults were collected for the bioassay at 24-h intervals.

### Insecticides

We tested four insecticides having different modes of action and belonging to two chemical classes: spinosyns (Nicotinic acetylcholine receptor allosteric modulators) and diamides (Ryanodine receptor modulators) (Table 1). These plant protection products are currently used in various crops (including tomato) worldwide and the two spinosyns are also authorized for organic farming. The highest recommended rates for tomato crops were used in our experiments (See Table 1 for more details). All the pesticides were stored and applied following their label guidelines. Clean 40-cm high potted tomato plants were sprayed with the insecticide solutions using a 1L power-pack aerosol hand sprayer (Matabi^®^, Antzuola, Guipuzcoa, Spain), and the nozzle of the sprayer was directed toward the plants from a distance of 0.5 m until runoff. This resulted in a complete and uniform wetting of the young tomato plants, using approximately 0.25L of insecticide solution per plant. Treated plants were then left to dry for 2 hours under laboratory conditions to obtain tomato plants uniformly covered with corresponding insecticide fresh dry residues. Afterwards, the top-part of the plant (about 17 cm) was cut and placed into a bioassay isolator made up of two superposed plastic cups, see Biondi et al. [6] for a thorough description of the experimental arena.

**Table 1:** Tested insecticides.

Active ingredient (a.i.)	Trade name	Field rate (a.i.%)*	Chemical family	Target pests	Mode of action
**spinetoram**	Radiant^®^ SC	731mL/ha (12%)	Spinosyn	Moths, thrips, leafminers	Ingestion and contact. Disruption of nicotinic/gamma amino butyric acid-gated chloride channels
**spinosad**	Laser^®^	25mL/hL (48%)	Spinosyn	Moths, thrips, leafminers, fruit flies	Ingestion and contact. Disruption of nicotinic/gamma amino butyric acid-gated chloride channels
**cyantraniliprole**	Exirel^TM^	1.5L/ha (10.2%)	Diamide	Lepidoptera, Coleoptera, leafminers, fruit flies, aphids, psyllids, thrips, whiteflies	Ingestion. Ryanodine receptors modulator
**chlorantraniliprole**	Altacor^®^	12g/hl (35%)	Diamide	Coleoptera, Diptera, Lepidoptera	Ingestion. Ryanodine receptors modulator

* The maximum label rate for tomato with a volume of 1000L ha^-1^of insecticide solutions were applied.

### Acute toxicity assessment at different temperatures

Newly emerged (0/24-h old) *B*. *nigricans* adults were exposed to dried pesticide residues on tomato leaves for three consecutive days. Five *B*. *nigricans* females and five males were released into each arena containing a treated tomato young stem (see previous section) and provided with untreated droplets of honey. Pesticide exposure assay was carried out at three constant temperatures, i.e. 25°C, 35°C and 40°C, in climatic cabinets set at fixed relative humidity (60%) and photoperiod (14L:10D). Five to six replicates per temperature, per tested insecticide and water treated control were carried out. The numbers of survived and dead parasitoids were recorded after three days of exposure. Parasitoids were considered dead when they remained immobile after being stimulated with a fine paintbrush.

### Impact on parasitism activity and female fecundity at different temperatures

Adult wasps, which survived the three-day exposure period to the insecticides in the acute toxicity trial, were grouped in couples (one male and one female) and the sublethal effects on reproduction and host-killing activity were evaluated at the regular constant temperature of 25°C. Preliminary trials showed that parasitoids exposed to untreated tomato leaves, for three consecutive days at the constant temperature of 40°C do not lay any egg in the following three days. Therefore, the sublethal effect assessment was carried out only for those parasitoids surviving the exposures at the normal and high temperatures (25°C and 35°C). Fourteen to 21 couples of survived parasitoids were tested per insecticide and per temperature.

Each couple was released into a modified Petri dish (similar to that used for parasitoid rearing, see description above) together with five newly molted fourth instars larvae of *T*. *absoluta*. Honey droplets were offered to the wasps on the inner surface of the lids using a fine paintbrush. In addition, young clean tomato leaflets were used to feed the moth larvae. For each female parasitoid, daily checks were carried out to count the number of laid eggs (as proxy of parasitoid reproduction) and the number of killed larvae (as proxy of parasitoid biological control services). Killed and/or parasitized larvae usually bear parasitoid eggs and/or necrotic spots developed after parasitoid stinging activity and/or feeding tubes built by the female for host-feeding (see Biondi et al. [49]). *Bracon nigricans* couples were transferred daily and for three days into new similar Petri dishes containing five *T absoluta* larvae. *Tuta absoluta* larvae exposed to the survived *B*. *nigricans* adults were then reared until adult emergence at 25±1°C, 50±10% R.H and 14L:10D. The number of adults emerged and the sex-ratio of the progeny were recorded. Very few and no females survived to spinetoram and spinosad exposure, respectively (see the results section). As a consequence, the sublethal effect assessment on survivors was not possible for these two insecticides.

### Data analyses

Raw datasets were tested for normality and homogeneity of variance using the Kolmogorov-Smirnov D test and the Cochran test, respectively and, when necessary, were transformed as follows: percentages of survival were subjected to arcsin square root transformation and number of laid eggs and killed host larvae to log (x+1) transformation. For the lethal and sublethal trials, we tested the effects of the insecticide (factor *treatment*) and of exposure temperature (factor *temperature*) and potential interactions between these two factors on (i) the percentages of parasitoids found dead after 3 days of exposure to the various insecticides, (ii) the cumulative numbers of eggs laid and killed host larvae during the three days following the insecticide exposure. For this, we used a multifactorial ANOVA. Subsequently, additional one-way ANOVA followed by LSD post hoc tests for multiple comparisons inside the three temperatures and the five treatments sub-datasets were carried out.

For each tested pesticide and temperature a *Reduction coefficient E*_*x*_ was calculated according to Biondi et al. [6]. For this, the female corrected mortality (*E*_*mx*_) (i.e., the % of mortality after the exposure to the pesticide residues for three days corrected for control mortality), and the percentages of reduction of female offspring (*E*_*fx*_) (calculated as the difference of eggs laid by the females exposed for three days to the insecticide residues and by the control females) were included in the formula. Finally, two demographic growth indices were estimated: the *Doubling time* (*DT*), that represents the time required for a given population to double in size, and the *Intrinsic rate of increase* (*r*_*m*_), a measure of the times the population will multiply itself per unit of time [49]. To get these values, *E*_*mx*_ and *E*_*fx*_ were incorporated into an age-structured *Leslie matrix* model [31,50] that consists of a matrix with the *B*. *nigricans* life-history elements (such as age-specific female survival and female offspring production) previously developed for a control population in similar experimental conditions [49].

## Results

### Acute toxicity

**Fig 1:**
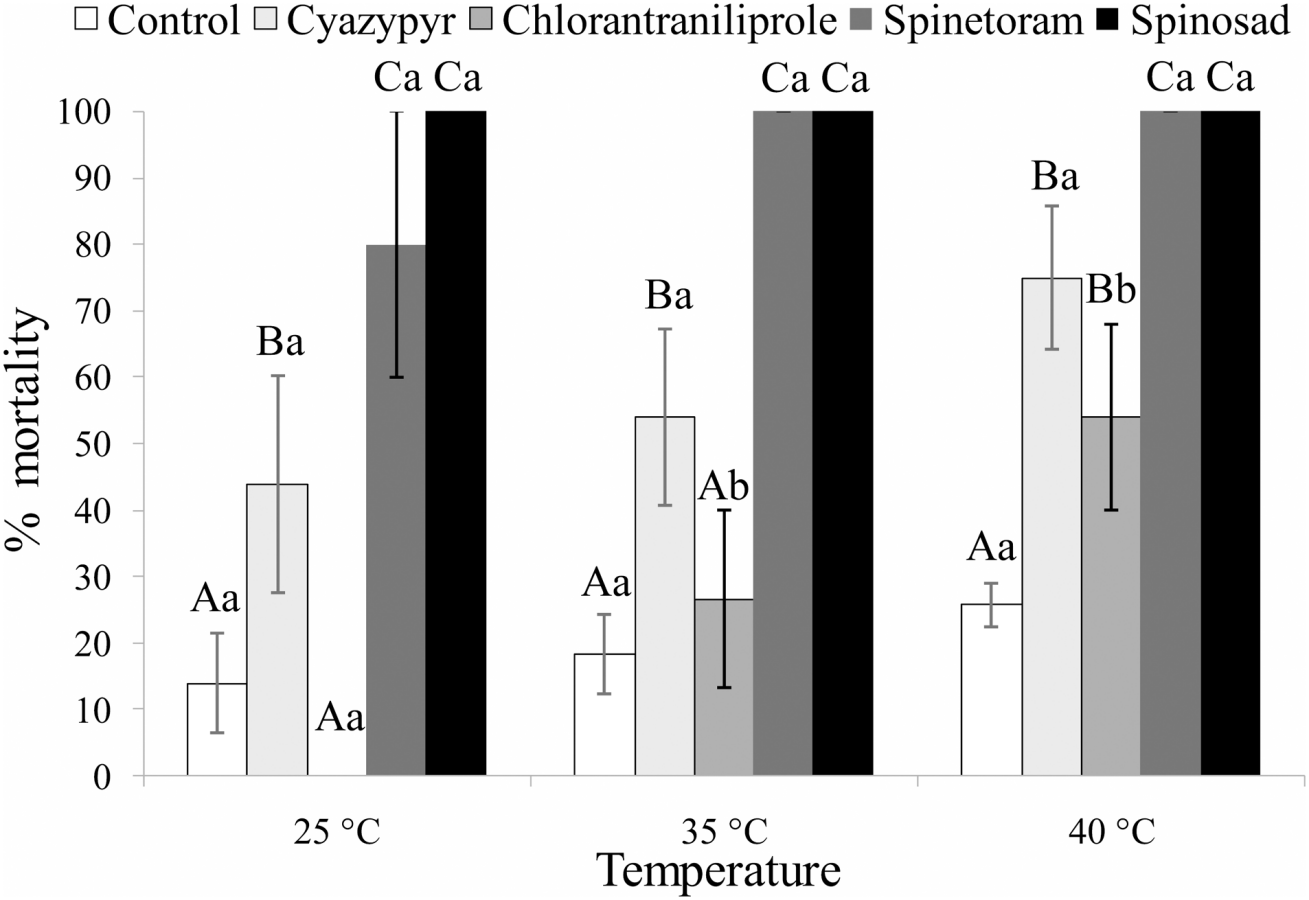
Lethal effects. Mean percentages (± SEM) of survival of *Bracon nigricans* adults when exposed for three days to insecticide residues at three constant temperatures. Columns bearing the same letter (upper case letters: within the same temperature regime; lower case letters: within the same tested insecticide) are not significantly different (*P*>0.05; ANOVA with LSD post hoc test for multiple comparisons).

The factor insecticide and the factor temperature significantly affected the survival of the adult parasitoids. However, the interaction of these two factors did not affect this parameter significantly (Fig 1). The statistical results of the multifactorial ANOVA are summarized in Table 2A. Mortality varied significantly among insecticides, within each tested temperature. At 25°C and 35°C the two spinosyns caused the highest mortality rates (almost total and total for spinetoram and spinosad, respectively), and cyantraniliprole mortality was significantly higher than control and chlorantraniliprole (25°C: *F*_*4*,*20*_ = 11.577; *P*<0.001; 35°C: *F*_*4*,*24*_ = 18.053; *P*<0.001). At the very high temperature, cyantraniliprole and chlorantraniliprole also caused significantly higher mortality than in the control, but significantly lower than the two spinosyns (*F*_*4*,*20*_ = 18.374; *P*<0.001) (Fig 1).

**Table 2:** Statistics from the multifactorial ANOVA used to analyze (A) the percentages of parasitoids found dead after 3 days of exposure to insecticides (mortality), (B) the total numbers of eggs laid (reproduction) and (C) the numbers of host larvae killed (biocontrol) by each *Bracon nigricans* female during the three days following the insecticide exposure. *P* values indicates in bold are those significant at <0.05 level.

Source of variation	Degrees of freedom	F	p-value
**A: Mortality**			
*Insecticide*	4, 68	43.989	**< 0.001**
*Temperature*	2, 68	6.793	**0.002**
*Insecticide X Temperature*	8, 68	1.077	0.390
**B: Reproduction**			
*Insecticide*	2, 88	0.595	0.094
*Temperature*	1, 88	0.042	0.838
*Insecticide X Temperature*	2, 88	2.387	**0.035**
**C: Biocontrol services**			
*Insecticide*	2, 88	6.912	**0.002**
*Temperature*	1, 88	3.916	**0.041**
*Insecticide X Temperature*	2, 88	1.216	0.301

The mortality rates registered for untreated control (*F*_*2*,*14*_ = 1.108; *P* = 0.358), cyantraniliprole (*F*_*2*,*14*_ = 1.392; *P* = 0.281), spinosad (*F*_*2*,*14*_ = 1.235; *P* = 0.999) and spinetoram (*F*_*2*,*14*_ = 1.201; *P* = 0.359) did not vary significantly among the three temperatures. However, in the case of chlorantraniliprole (*F*_*2*,*14*_ = 3.941; *P* = 0.038) we registered an increasing temperature-related trend in acute toxicity, with survival rates significantly lower at 40°C compared to the two lower temperatures (Fig 1).

### Sublethal effects on reproduction and biocontrol activity

**Fig 2:**
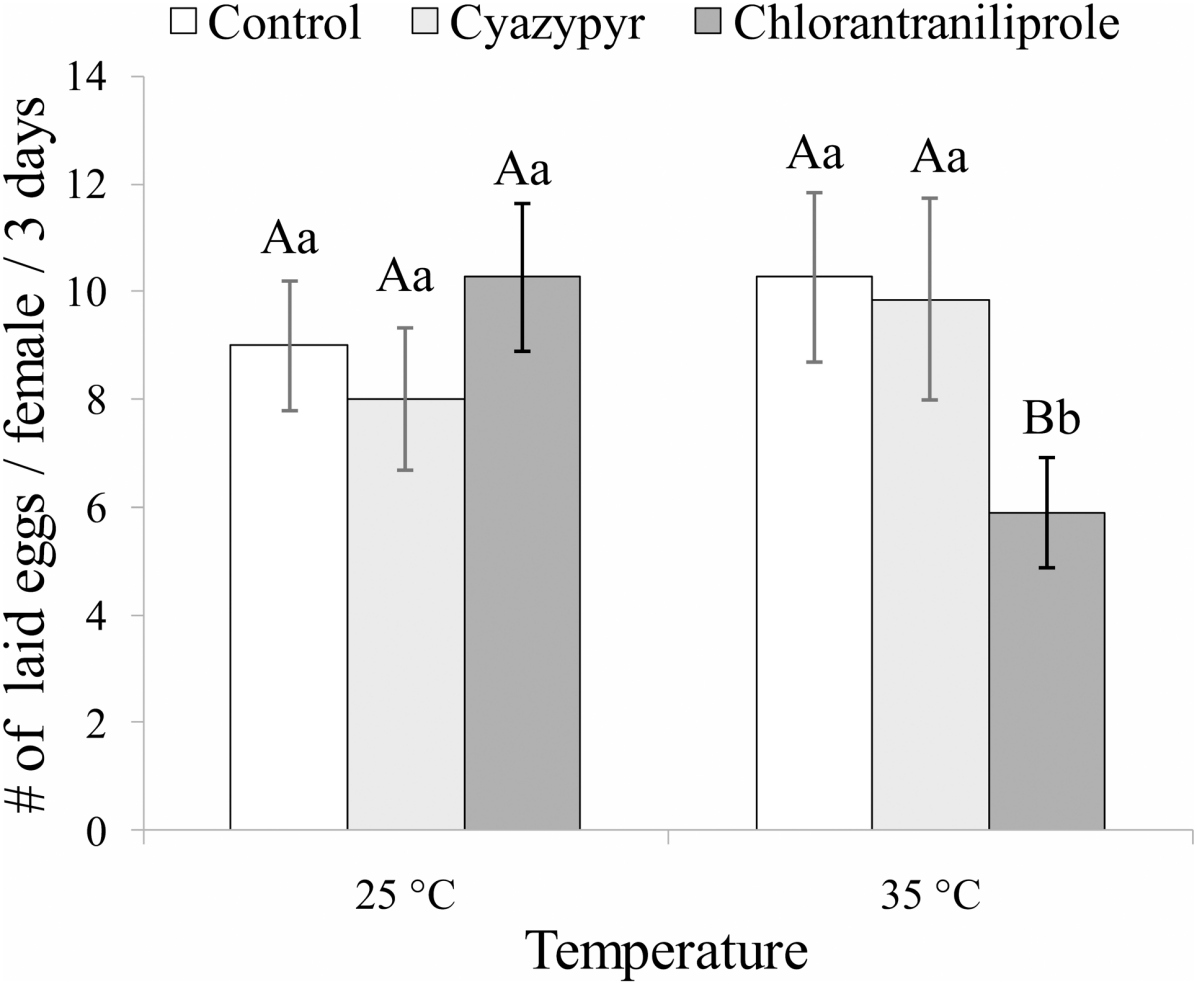
Sublethal effects on reproduction. Means (± SEM) of number of eggs laid in three days by each tested *Bracon nigricans* female previously exposed for three days to residues at three constant temperatures. Columns bearing the same letter (upper case letters: within the same temperature regime; lower case letters: within the same tested insecticide) are not significantly different (*P*>0.05; ANOVA with LSD post hoc test for multiple comparisons).

The statistical results of the multifactorial ANOVA on the number of laid eggs are summarized in Table 2B. The temperature factor did not significantly influence the number of laid eggs, the insecticide factor was not far from significance (0.10 > *P* > 0.05), while the interaction between these two factors significantly influenced the number of eggs laid by *B*. *nigricans* females (Fig 2). Indeed, only in the case of chlorantraniliprole (*F*_*1*,*34*_ = 6.838; *P* = 0.013) the number of eggs laid was significantly lower than those of the control and of cyantraniliprole at 35°C, but it was not at 25°C. Whereas, no significant differences on this trait between the two temperatures were recorded for control (*F*_*1*,*35*_ = 0.208; *P* = 0.651), and cyantraniliprole-exposed female wasps (*F*_*1*,*31*_ = 0.010; *P* = 0.972) (Fig 2). As a consequence, within the temperature sub-datasets the treatment was not significant at 25°C (*F*_*2*,*44*_ = 0.875; *P* = 0.424), but it was significant at 35°C (*F*_*2*,*51*_ = 4.133; *P* = 0.022) (Fig 2).

**Fig 3:**
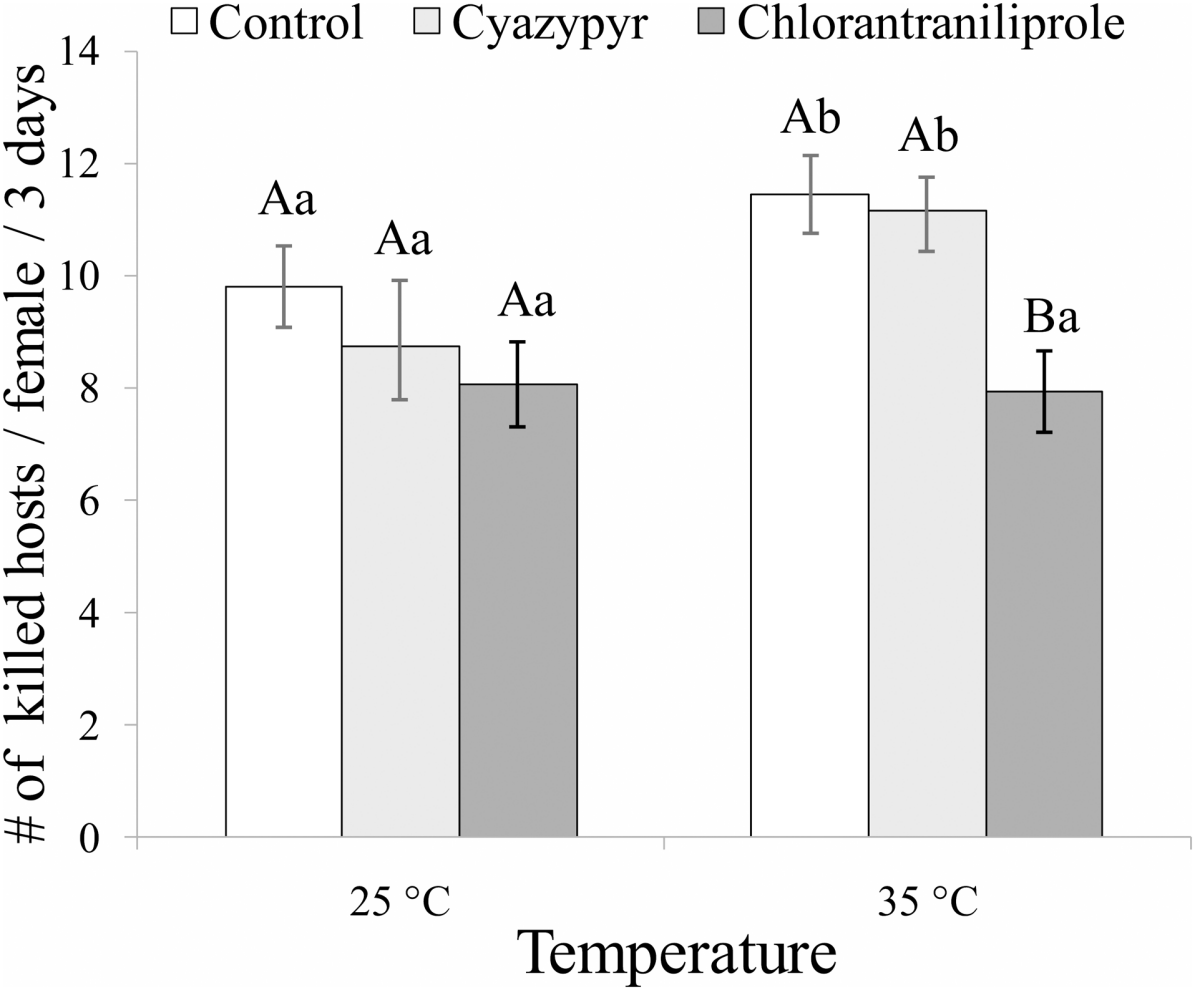
Sublethal effects on biocontrol activity. Means (± SEM) of number of host larvae killed in three days by each tested *Bracon nigricans* female previously exposed for three days to residues at three constant temperatures. Columns bearing the same letter (upper case letters: within the same temperature regime; lower case letters: within the same tested insecticide) are not significantly different (*P*>0.05; ANOVA with LSD post hoc test for multiple comparisons).

Insecticide and temperature factors, but not their interaction, affected the host killing activity (Fig 3). The statistical results of the multifactorial ANOVA on the number of killed larvae are summarized in Table 2C. No significant differences in host-killing among the treatments were noticed at 25°C (*F*_*2*,*45*_ = 1.1443; *P* = 0.247), but it was not the case at 35°C (*F*_*2*,*50*_ = 6.965; *P*<0.002) (Fig 3). The number of killed larvae by control female wasps (*F*_*1*,*30*_ = 4.383; *P* = 0.045) and by those exposed to cyantraniliprole (*F*_*1*,*32*_ = 4.171; *P* = 0.048), but not by those exposed to chlorantraniliprole (*F*_*1*,*28*_ = 0.016; *P* = 0.900), was significantly higher after the exposure to insecticide residues at 35°C than at 25°C (Fig 3).

### Reduction coefficient (*Ex*) and Demographic growth parameters

The highest reduction coefficients (*E*_*x*_) were obtained with the two tested spinosyns. Indeed, due to the very high acute toxicity of these insecticides, we estimated an *E*_*x*_ of 100% after 3 days-exposure to both spinosad and spinetoram at the 3 tested temperatures, with the exception of spinetoram at 25°C which caused an *E*_*x*_ of 76.74% (Table 3). However, it caused a delay in population size doubling time (*DT*) of 598.6 d as well as a very high reduction of the parasitoid intrinsic rate of increase (*r*_*m*_) which was considerably lower compared to the control (Table 3). No demographic indexes were generated for spinosad at 40°C and for spinetoram at 35°C and 40°C because all individuals died after residual contact with dry insecticide residues.

**Table 3:** Reduction coefficient *E*_*x*_ [6], Doubling time (*DT*) and Intrinsic rate of increase (*r*_*m*_) [35] estimated for *Bracon nigricans* adults exposed to the four tested insecticides at three constant temperatures and for the control parasitoid population.

Treatment	Reduction coefficient (*E*_*x*_)			Doubling time (*DT*)			Intrinsic rate of increase (*r*_*m*_)		
	25°C	35°C	40°C	25°C	35°C	40°C	25°C	35°C	40°C
Control	-	-	-	13.82	13.82	13.82	0.052	0.052	0.052
Cyantraniliprole	35.74	46.07	66.29	21.81	23.59	50.98	0.032	0.029	0.014
Chlorantraniliprole	0.00	48.45	47.98	13.82	24.86	24.59	0.052	0.028	0.028
Spinetoram	76.74	100.00	100.00	620.40	-	-	0.001	-	-
Spinosad	100.00	100.00	100.00	-	-	-	-	-	-

The *E*_*x*_ due to cyantraniliprole increased with temperature and it reached its maximum value (66.29) at 40°C (Table 3). *DT* increased from 8 d at 25°C, to 9.8 d at 35°C and to 37.2 at 40°C. A joint effect of pesticide and temperature was also evident when estimating the wasp intrinsic rate of increase which was lower compared to the untreated control at the 3 tested temperatures, with the lowest value recorded at 40°C (Table 3). The exposure to chlorantraniliprole fresh residues at 25°C caused no effect on *B*. *nigricans* both in terms of *E*_*x*_, *DT* and *r*_*m*_. At 35°C and 40°C the combined side effects of insecticide exposure and temperature were almost identical with an *E*_*x*_ at 48%, a delay in *DT* of 11 d and a halved *r*_*m*_ (Table 3).

## Discussion

The outcomes of the study of the interaction between a natural stressor, i.e. high temperatures, and the residual contact with dry insecticide residues were studied in the laboratory toward adults of an insect parasitoid. We found that lethal and sublethal effects of two out of the four studied insecticides can vary among temperature regimes. In particular, although the two spinosyns and cyantraniliprole exerted the same acute toxicity levels among the three tested temperatures, for chlorantraniliprole the acute toxicity and the effects on the reproduction were significant only at 40°C and 35°C, respectively. Moreover, *B*. *nigricans* females exposed for three days at 35°C to untreated and cyantraniliprole-treated tomato leaves showed an increased host-killing activity. This can be probably due to the natural need of the females to recover by assimilating more fluids and energy after the exposure to the high temperature, thus increasing the host handling time prior to egg laying, but this should be specifically tested via behavioral observations. Increasing host handling time was documented for the pteromalid parasitoid *Theocolax elegans* (Wetwood), where the handling time of its host *Sitophilus oryzae* L. (Coleoptera: Curculionidae) was inversely proportional to temperature within the range of 17–39°C with the instantaneous search rate with increasing temperature [51]. As pointed out by Amat et al. [52] for the ichneumonid wasp *Venturia canescens* (Gravenhorst), one possible explanation is related to the exploitation strategy and time allocation changes in relationship with temperature regime. A positive correlation between toxicity and growing temperature was recorded in a number of studies conducted on insects, due to an increased uptake of chemicals at higher temperatures [10].

Cyantraniliprole was slightly toxic to *B*. *nigricans* adults in terms of acute toxicity at the three temperatures, while it did not cause any sublethal effects in egg-laying and host-killing activities. However, acute toxicity was responsible of delays in population doubling time of 8, 10 and 32 days at 25, 35 and 40°C, respectively. To the best of our knowledge, this is one of the first reports on non-target toxicity assessment for this novel chemical towards beneficial insects. Amarasekare and Shearer [53] assessed lethal and sublethal effects of cyantraniliprole on two lacewing species and found no significant effects on larval mortality while causing significant adult mortality. Conversely, cyantraniliprole was selective to three predatory mites as reported by Reis et al. [54]. Similarly, no negative impact of this insecticide was detected on the populations of pirate bugs *Orius insidiosus* (Say) and *O*. *pumilio* (Champion) (Hemiptera: Anthocoridae), predators of thrips in pepper crops [55]. Likewise, minimum adverse effects of this newly developed insecticide were reported on *Tamarixia triozae* (Burks) (Hymenoptera: Eulophidae) an important parasitoid of the potato or tomato psyllid, *Bactericera cockerelli* (Sulc) (Hemiptera: Triozidae), a serious pest of many solanaceous vegetables [56]. Supported by these data, sprayings of this insecticide could be considered in IPM programs in tomato. However, further studies on non-target toxicity on auxiliary fauna associated with tomato crops are still needed to advance our understanding on its possible impact on both lepidopterous pests and their natural enemies. Moreover, our results do support the need to include natural stressors, such as temperature, and the effects at the demographic scale as two key factors of these future studies. Indeed, the procedures for insecticide risk assessment should take into consideration the impact of abiotic factors and include demographic traits as a valuable tool in evaluating ecotoxicological profiles of intoxicants and their long term hidden effects on beneficial insects, as well as the possibilities of post treatment recovery [57].

The other diamide, chlorantraniliprole, showed to be the safest among the four tested insecticides. This result agrees with most of those obtained studying predatory insects, including on tomato. Biondi et al. [6] found no negative effects of chlorantraniliprole exposure on *Orius laevigatus* (Fieber). Martinou et al. [58] showed that in the laboratory, chlorantraniliprole has low non-target effects on *Macrolophus pygmaeus* (Rambur) (Hemiptera: Miridae) with minimum adverse behavioral side effects mainly through the decrease in plant feeding by this zoophytophagous insect. Similarly, Zhang et al. [59] studied the impact of this insecticide on another mirid predator, *Cyrtorhinus lividipennis* (Reuter), and concluded that it has no significant impact on its behavioral response and predation capability. In case of lacewings (Neuroptera: Chrysopidae) *Chrysoperla johnsoni* (Henry) and *Chrysoperla carnea* (Stephens), no significant mortality was recorded on larvae when exposed to chlorantraniliprole, while high mortality and reproduction reduction of adults was recorded [33,53]. Nevertheless, contrasting results on the efficiency of this novel insecticide is reported for *T*. *absoluta* worldwide. Reduction in fruit damage between 75% and 85% for foliar applications and 82% for soil applications in Chilean tomato cultivations [60], and strong metabolic resistance phenomena have been recently described for a South European population of *T*. *absoluta* [46]. Overall, if our results at different temperature regimes will be confirmed by further studies on other non-target species, it can be stated that chlorantraniliprole can be safely included into IPM schemes in tomato crops, however caution should be paid when pollination with bumblebees is expected. Smagghe et al. [61] found that chlorantraniliprole affects the *Bombus terrestris* L. (Hymenoptera: Apidae) drone production by suppressing reproduction in worker bumblebees following treated pollen intake.

The results obtained for the two spinosyns agree with what was already elucidated for the hymenopteran parasitoids that were shown to be very susceptible to spinosad (see Biondi et al. [2], for a thorough review). However, in the case of spinetoram this is one of the first reports of non-target toxicity toward insect parasitoids of agricultural pests. Hernandez et al. [62] studied the effect of the field recommended rate of spinetoram on the longevity of two parasitoid species of Diptera agromizid, *Ganaspidium nigrimanus* (Kieffer) (Hymenoptera: Figitidae) and *Neochrysocharis formosa* (Westwood) (Hymenoptera: Eulophidae), the latter also a *T*. *absoluta* parasitoid [44]. The survivorship of these species decreased significantly when individuals were exposed to spinetoram either topically, on pepper leaves, or through feeding on a spinoteram-contaminated food source. Therefore, these results suggest that this novel spinosyn, as its predecessor spinosad, has not a favorable toxicological profile toward insect parasitoids. Nevertheless, some studies reported high cross insecticide resistance in populations of *T*. *absoluta* in relationship with the overuse of spinosad [63,64]. Therefore, it has been suggested avoiding spinosad sprays against the tomato borer, which would be useful in terms of resistance management for this pest and adverse side effects on its natural enemies, including *B*. *nigricans*.

Overall, the results of this study can be very useful for choosing the insecticide with lower risks towards parasitoids that provide biocontrol services in tomatoes, particularly in those environments where high temperatures are common. The two diamide insecticides, chlorantraniliprole and cyantraniliprole were demonstrated to have a safer profile towards this wasp species compared to the two tested spinosyns. Nevertheless, we showed that for some insecticides, including the very new diamide cyantraniliprole, high temperatures can interfere with the non-target toxicity of compounds used in crop protection.

## Supporting Information

S1 Dataset Lethal and sublethal effects raw data. Mortality, number of eggs laid and number of hosts killed in three days by each tested *Bracon nigricans* female previously exposed for three days to residues at three constant temperatures.
(XLSX)
